# Long‐term safety and efficacy of the sodium–glucose cotransporter 2 inhibitor, tofogliflozin, added on glucagon‐like peptide‐1 receptor agonist in Japanese patients with type 2 diabetes mellitus: A 52‐week open‐label, multicenter, post‐marketing clinical study

**DOI:** 10.1111/jdi.13066

**Published:** 2019-05-28

**Authors:** Yasuo Terauchi, Hisataka Fujiwara, Yuji Kurihara, Hideki Suganami, Masahiro Tamura, Masayuki Senda, Ryoji Gunji, Kohei Kaku

**Affiliations:** ^1^ Yokohama City University School of Medicine Yokohama Japan; ^2^ Post Marketing Surveillance Department Kowa Company, Ltd. Tokyo Japan; ^3^ Clinical Data Science Department Kowa Company, Ltd. Tokyo Japan; ^4^ Post‐Authorization Regulatory Studies Sanofi K.K. Tokyo Japan; ^5^ Department of Medicine Kawasaki Medical School Kurashiki Japan

**Keywords:** Glucagon‐like peptide‐1 receptor, Sodium–glucose cotransporter 2 inhibitor, Type 2 diabetes mellitus

## Abstract

**Aims/Introduction:**

Tofogliflozin is a potent and highly selective sodium–glucose cotransporter 2 inhibitor that is currently used to treat patients with type 2 diabetes mellitus. The aim of the present study was to evaluate the safety and efficacy of tofogliflozin add‐on to glucagon‐like peptide‐1 (GLP‐1) receptor agonist monotherapy.

**Materials and Methods:**

In this 52‐week, prospective, multicenter, single arm, post‐marketing clinical study, Japanese patients who had already been receiving GLP‐1 receptor agonist monotherapy for ≥8 weeks, glycated hemoglobin ≥7.0 and <10.5%, and body mass index ≥18.5 and <35.0 kg/m^2^ were enrolled. Tofogliflozin 20 mg was orally administered once daily for 52 weeks with GLP‐1 receptor agonist. Primary end‐points were safety and change in glycated hemoglobin from baseline to week 52. Safety was assessed on the basis of the adverse events. Changes from baseline in fasting plasma glucose, bodyweight, blood pressure, uric acid and lipid parameters were assessed as secondary efficacy end‐points.

**Results:**

Of the 67 patients enrolled, 63 patients completed the study. Overall, 26 adverse drug reactions occurred in 17 patients (25.4%). Adverse drug reactions with a frequency of two or more patients (3.0%) were constipation, thirst, dehydration and pollakiuria. Hypoglycemia (*n* = 1) was limited. With the addition of tofogliflozin to GLP‐1 receptor agonist, the subsequent mean (standard deviation) reduction in glycated hemoglobin was −0.6% (1.0%; *P *<* *0.0001). Fasting plasma glucose, bodyweight and blood pressure were significantly improved.

**Conclusions:**

Tofogliflozin add‐on to GLP‐1 receptor agonist monotherapy is an effective treatment option with an acceptable safety profile.

## Introduction

Tofogliflozin is a potent and highly selective sodium–glucose cotransporter 2 (SGLT2) inhibitor^1^, ^2^, ^3^, and is currently used for the treatment of patients with type 2 diabetes mellitus in Japan^4^, ^5^. SGLT2 inhibitors exert their antidiabetic effects by inhibiting SGLT2, which is localized in the proximal renal tubule and is responsible for approximately 90% of glucose reabsorption in the kidney^6^. Suppression of renal glucose reabsorption and an increase in urinary glucose excretion results in decreased blood glucose levels^7^.

Once daily oral administration of tofogliflozin for 24–52 weeks showed clinically relevant improvements in glycemic control associated with weight loss in phase III and long‐term clinical trials^8^, ^9^, ^10^. In these trials, tofogliflozin was well tolerated, and most adverse drug reactions (ADRs) were mild or moderate in severity. Even in cases when tofogliflozin was added to oral antidiabetic agents, the efficacy and safety were consistent regardless of the background therapy^10^. The adverse events (AEs) of tofogliflozin observed in the clinical trials were hyperketonemia, dry mouth and pollakiuria, which are known as class effects of SGLT2 inhibitors^11^. The post‐marketing study of tofogliflozin in elderly patients aged ≥65 years showed that the incidence of ADRs was similar to those observed in preapproval trials with no additional special concerns^12^. The risk of hypoglycemia was low when tofogliflozin was administered as monotherapy^13^, ^14^.

Because of the insulin‐independent mode of action, a SGLT2 inhibitor can be used in combination with any class of antidiabetic agents with complementary mechanisms of action. In particular, glucagon‐like peptide‐1 (GLP‐1) receptor agonists would be a favorite candidate for combination with a SGLT2 inhibitor, as the available GLP‐1 receptor agonists show superiority to other antidiabetic agents in glycated hemoglobin (HbA1c) reduction, weight loss and blood pressure reduction, as well as SGLT2 inhibitors^15^. Furthermore, it is noted that the risk of hypoglycemia in both classes of the compound is limited^16^.

In the present study, we report the 52‐week safety and efficacy of tofogliflozin when administered to patients with type 2 diabetes mellitus who were treated with GLP‐1 receptor agonist monotherapy, but were controlled inadequately.

## Methods

### Study design

This was a long‐term (52‐week), prospective, multicenter, single arm, post‐marketing clinical study to investigate the safety and efficacy of tofogliflozin when co‐administered to patients with type 2 diabetes mellitus treated with GLP‐1 receptor agonist. The study protocol, informed consent form and other relevant study documents were approved by the institutional review board at each site. All patients gave written informed consent before initiation of any study‐specific procedures. The study was carried out in accordance with the Guideline for Clinical Evaluation of Oral Hypoglycemic Agents^17^, the ethical principles originating in or derived from the Declaration of Helsinki, International Council on Harmonization Good Clinical Practice Guidelines, Good Post‐Marketing Study Practice, and locally applicable laws and regulations.

### Participants and treatment

Patients with type 2 diabetes mellitus already receiving GLP‐1 receptor agonist monotherapy for ≥8 weeks were enrolled. The inclusion criteria were age 20–75 years; HbA1c ≥7.0 and <10.5%; body mass index (BMI) ≥18.5 kg/m^2^ and <35.0 kg/m^2^; and no change of the dose and regimen of GLP‐1 receptor agonist for ≥8 weeks before screening and during the study. Key exclusion criteria were type 1 diabetes; fasting plasma glucose (FPG) ≥270 mg/dL; unstable proliferative diabetic retinopathy, or other rapidly progressive diabetic retinopathy or macular edema possibly requiring treatment; history of metabolic acidosis, including diabetic acidosis within 1 year before screening; myocardial infarction, stroke or heart failure that required hospitalization within 6 months before screening; start of treatment with an anti‐obesity drug during the 3 months before screening; or severe hepatic or renal disorder. During the study, antidiabetic agents other than GLP‐1 receptor agonist were prohibited. Tofogliflozin 20 mg tablet was orally administered once daily either before or after breakfast for 52 weeks.

Safety was assessed according to the incidence and severity of AEs, clinical laboratory test (hematology, biochemistry and urinalysis), vital signs, and 12‐lead electrocardiogram. The primary efficacy end‐point was change in HbA1c from baseline to week 52. Secondary efficacy end‐points were changes from baseline to week 52 in FPG; bodyweight; systolic blood pressure (SBP); diastolic blood pressure (DBP), uric acid; and lipid parameters including total cholesterol (TC), high‐density lipoproteins cholesterol (HDL‐C), low‐density lipoprotein cholesterol (LDL‐C), triglyceride (TG), free fatty acid (FFA) and non‐HDL cholesterol. For assessment of efficacy, HbA1c, FPG, bodyweight, SBP and DBP were evaluated at screening, and week 0, 4, 8, 12, 16, 24, 32, 40 and 52. C‐peptide, insulin, proinsulin, glucagon, glycated albumin, proinsulin/insulin ratio and other clinical tests were assessed at screening and week 0, 4, 12, 24, 32, 40 and 52.

### Statistical analysis

Adverse events and ADRs were categorized according to the Medical Dictionary for Regulatory Activities/Japanese edition version 20.0. The safety analysis set was defined as patients who took at least one dose of tofogliflozin and had a safety measurement. Efficacy was analyzed in the full analysis set, which included all patients who received at least one dose of tofogliflozin, and had a baseline (week 0) and at least an efficacy measurement after the first treatment. Changes of continuous variables from baseline were summarized using the descriptive statistics, including mean and standard deviation (SD).

During efficacy assessment, changes of variables from baseline to end of treatment were compared using the one sample *t*‐test. For efficacy assessment, adjustment of multiplicity was not investigated because of the limited number of participants. The significance level was set at 5%, and the interval estimation used a two‐sided 95% confidence interval. Missing data at week 52 were imputed with the last observation carried forward (LOCF) method, and the change from baseline to week 52 was calculated using LOCF data. A sample size of 65 was selected according to the Guideline for Clinical Evaluation of Oral Hypoglycemic Agents^17^. SAS 9.2 or its updated version (SAS Institute Japan Ltd., Tokyo, Japan) was used for the statistical analyses.

## Results

### Patient disposition and characteristics

The present study was carried out from August 2015 to July 2017 at 12 sites in Japan (Table S1). Of the 67 patients registered, 63 patients completed the 52‐week treatment period with the study drug after exclusion of consent withdrawal (*n* = 3) and the investigator's decision (*n* = 1; Figure S1). The safety analysis set and full analysis set population comprised 67 patients. Table 1 summarizes the patient demographics and baseline characteristics. Of the 67 patients, 67.2% were men and 32.8% women. The mean age was 54.8 years (SD 8.5 years), and nine (13.4%) patients were aged ≥65 years. The mean BMI was 27.0 kg/m^2^ (SD 3.2 kg/m^2^). All patients received liraglutide as a GLP‐1 receptor agonist. The mean duration of diabetes was 9.6 years (SD 5.5 years). The mean baseline HbA1c and FPG were 8.6% (SD 1.0%) and 190.7 mg/dL (SD 39.0 mg/dL), respectively. All patients had concomitant diseases, in which 70.1% of patients had hyperlipidemia and 55.2% had hypertension. The mean duration of exposure and adherence of tofogliflozin were 347.0 days (SD 68.2 days; range 29–372 days) and 99.1% (2.4%; range 83.3–100%), respectively.

**Table 1:** Patient demographics and baseline characteristics (safety analysis set)

	*n* = 67
Age (years)	54.8 ± 8.5
Age category (years), *n* (%)	
<65	58 (86.6)
≥65	9 (13.4)
Sex	
Male, *n* (%)	45 (67.2)
Female, *n* (%)	22 (32.8)
Bodyweight (kg)	73.16 ± 10.33
BMI (kg/m^2^)	26.99 ± 3.20
BMI category (kg/m^2^), *n* (%)	
<25	19 (28.4)
≥25 to <30	36 (53.7)
≥30	12 (17.9)
Duration of diabetes (years)	9.6 ± 5.5
HbA1c (%)	8.57 ± 1.04
HbA1c (%) category, *n* (%)	
<8.0	22 (32.8)
≥8.0	45 (67.2)
FPG (mg/dL)	190.7 ± 39.0
eGFR (30 mL/min/1.73 m^2^)	89.4 ± 21.9
eGFR category (30 mL/min/1.73 m^2^), *n* (%)	
≥45 to <60	6 (9.0)
≥60 to <90	26 (38.8)
≥90	35 (52.2)
Systolic blood pressure (mmHg)	130.0 ± 15.4
Diastolic blood pressure (mmHg)	81.3 ± 9.8
Concomitant disease, *n* (%)	67 (100.0)
Hypertension	
Yes, *n* (%)	37 (55.2)
No, *n* (%)	30 (44.8)
Hyperlipidemia	
Yes, *n* (%)	47 (70.1)
No, *n* (%)	20 (29.9)

Data presented as the mean ± standard deviation. BMI, body mass index; eGFR, estimated glomerular filtration rate; FPG, fasting plasma glucose; HbA1c, glycated hemoglobin; SD, standard deviation.

### Safety results

The incidences of AEs and tofogliflozin‐related ADRs by system organ class and preferred term in the present study are summarized in Table 2 (see also Table S2,S3). Overall, 103 AEs occurred in 38 (56.7%) patients and 27 ADRs in 17 (25.4%) patients. ADRs with a frequency of two or more patients (3.0%) were constipation, thirst, dehydration and pollakiuria. ADRs associated with excessive urination were pollakiuria (*n* = 3, 4.5%) and urine output increased (*n* = 1, 1.5%). Volume depletion‐related events occurred in five patients (7.5%): thirst (*n* = 4, 6.0%), dehydration (*n* = 3, 4.5%), brain stem infarction (*n* = 1, 1.5%) and hypotension (*n* = 1, 1.5%). Hypoglycemia, urinary tract infection and sclerema were reported in one patient each (1.5%). Most AEs were mild in intensity, and moderate ADRs were hypoglycemia, chronic gastritis and brain stem infarction. One serious AE (SAE), that is, brain stem infarction, was reported and this was considered to be related to the study drug. Most ADRs were observed within the first 12 weeks after treatment initiation. No deaths were reported. Two patients discontinued the study drug due to the following AEs: pollakiuria, hypotension, thirst and brain stem infarction in one patient, and asthenia in another patient. Significant changes were observed in some clinical laboratory (hematology, biochemistry and urinalysis) items (Table S4). The presence of urine ketone bodies (*n* = 1) and increased urine output (*n* = 1) were reported as ADRs in clinical laboratories. No notable changes in physical examination, vital signs or electrocardiogram were observed.

**Table 2:** Adverse events in system organ class and preferred term

System organ class Preferred term	All		ADR	
	*n* = 67		*n* = 67	
	No. events	*n* (%)	No. events	*n* (%)
Cardiac disorders	1	1 (1.5)	0	0 (0)
Extrasystoles	1	1 (1.5)	0	0 (0)
Eye disorders	1	1 (1.5)	0	0 (0)
Dacryoadenitis acquired	1	1 (1.5)	0	0 (0)
Gastrointestinal disorders	12	10 (14.9)	4	4 (6.0)
Abdominal pain upper	1	1 (1.5)	0	0 (0)
Chronic gastritis	1	1 (1.5)	1	1 (1.5)
Constipation	3	3 (4.5)	3	3 (4.5)
Dental caries	1	1 (1.5)	0	0 (0)
Diarrhea	1	1 (1.5)	0	0 (0)
Diverticulum intestinal	1	1 (1.5)	0	0 (0)
Gastritis	1	1 (1.5)	0	0 (0)
Gastroesophageal reflux disease	2	2 (3.0)	0	0 (0)
Large intestine polyp	1	1 (1.5)	0	0 (0)
General disorders and administration site conditions	6	6 (9.0)	5	5 (7.5)
Asthenia	1	1 (1.5)	1	1 (1.5)
Malaise	1	1 (1.5)	0	0 (0)
Thirst	4	4 (6.0)	4	4 (6.0)
Hepatobiliary disorders	1	1 (1.5)	0	0 (0)
Cholelithiasis	1	1 (1.5)	0	0 (0)
Infections and infestations	35	21 (31.3)	1	1 (1.5)
Conjunctivitis	1	1 (1.5)	0	0 (0)
Gastroenteritis	2	2 (3.0)	0	0 (0)
Influenza	2	2 (3.0)	0	0 (0)
Periodontitis	3	2 (3.0)	0	0 (0)
Sinusitis	1	1 (1.5)	0	0 (0)
Urinary tract infection	1	1 (1.5)	1	1 (1.5)
Viral upper respiratory tract infection	23	15 (22.4)	0	0 (0)
*Helicobacter pylori*‐associated gastritis	1	1 (1.5)	0	0 (0)
Oral herpes	1	1 (1.5)	0	0 (0)
Injury, poisoning and procedural complications	7	3 (4.5)	0	0 (0)
Animal bite	1	1 (1.5)	0	0 (0)
Laceration	1	1 (1.5)	0	0 (0)
Muscle rupture	1	1 (1.5)	0	0 (0)
Contusion	3	1 (1.5)	0	0 (0)
Post‐traumatic neck syndrome	1	1 (1.5)	0	0 (0)
Investigations	6	6 (9.0)	2	2 (3.0)
Blood creatine phosphokinase increased	1	1 (1.5)	0	0 (0)
Blood pressure increased	1	1 (1.5)	0	0 (0)
Blood triglycerides increased	1	1 (1.5)	0	0 (0)
Urine positive for white blood cells	1	1 (1.5)	0	0 (0)
Urine ketone bodies present	1	1 (1.5)	1	1 (1.5)
Urine output increased	1	1 (1.5)	1	1 (1.5)
Metabolism and nutrition disorders	6	6 (9.0)	4	4 (6.0)
Dehydration	4	4 (6.0)	3	3 (4.5)
Hypercholesterolemia	1	1 (1.5)	0	0 (0)
Hypoglycemia	1	1 (1.5)	1	1 (1.5)
Musculoskeletal and connective tissue disorders	5	4 (6.0)	0	0 (0)
Back pain	3	3 (4.5)	0	0 (0)
Flank pain	1	1 (1.5)	0	0 (0)
Osteoarthritis	1	1 (1.5)	0	0 (0)
Nervous system disorders	4	4 (6.0)	2	2 (3.0)
Brain stem infarction	1	1 (1.5)	1	1 (1.5)
Dizziness	1	1 (1.5)	0	0 (0)
Dysgeusia	1	1 (1.5)	1	1 (1.5)
Carotid arteriosclerosis	1	1 (1.5)	0	0 (0)
Psychiatric disorders	1	1 (1.5)	0	0 (0)
Insomnia	1	1 (1.5)	0	0 (0)
Renal and urinary disorders	3	3 (4.5)	3	3 (4.5)
Pollakiuria	3	3 (4.5)	3	3 (4.5)
Reproductive system and breast disorders	2	2 (3.0)	2	2 (3.0)
Balanoposthitis	1	1 (1.5)	1	1 (1.5)
Pruritus genital	1	1 (1.5)	1	1 (1.5)
Respiratory, thoracic and mediastinal disorders	7	6 (9.0)	0	0 (0)
Cough	1	1 (1.5)	0	0 (0)
Upper respiratory tract inflammation	5	4 (6.0)	0	0 (0)
Oropharyngeal pain	1	1 (1.5)	0	0 (0)
Skin and subcutaneous tissue disorders	4	3 (4.5)	2	1 (1.5)
Pustular psoriasis	1	1 (1.5)	0	0 (0)
Sclerema	2	1 (1.5)	2	1 (1.5)
Urticaria	1	1 (1.5)	0	0 (0)
Vascular disorders	2	2 (3.0)	2	2 (3.0)
Hypotension	1	1 (1.5)	1	1 (1.5)
Orthostatic hypotension	1	1 (1.5)	1	1 (1.5)

Medical Dictionary for Regulatory Activities/Japanese edition version 20.0. ADR, adverse drug reaction.

### Changes in efficacy‐related laboratory variables and vital signs

**Figure 1:**
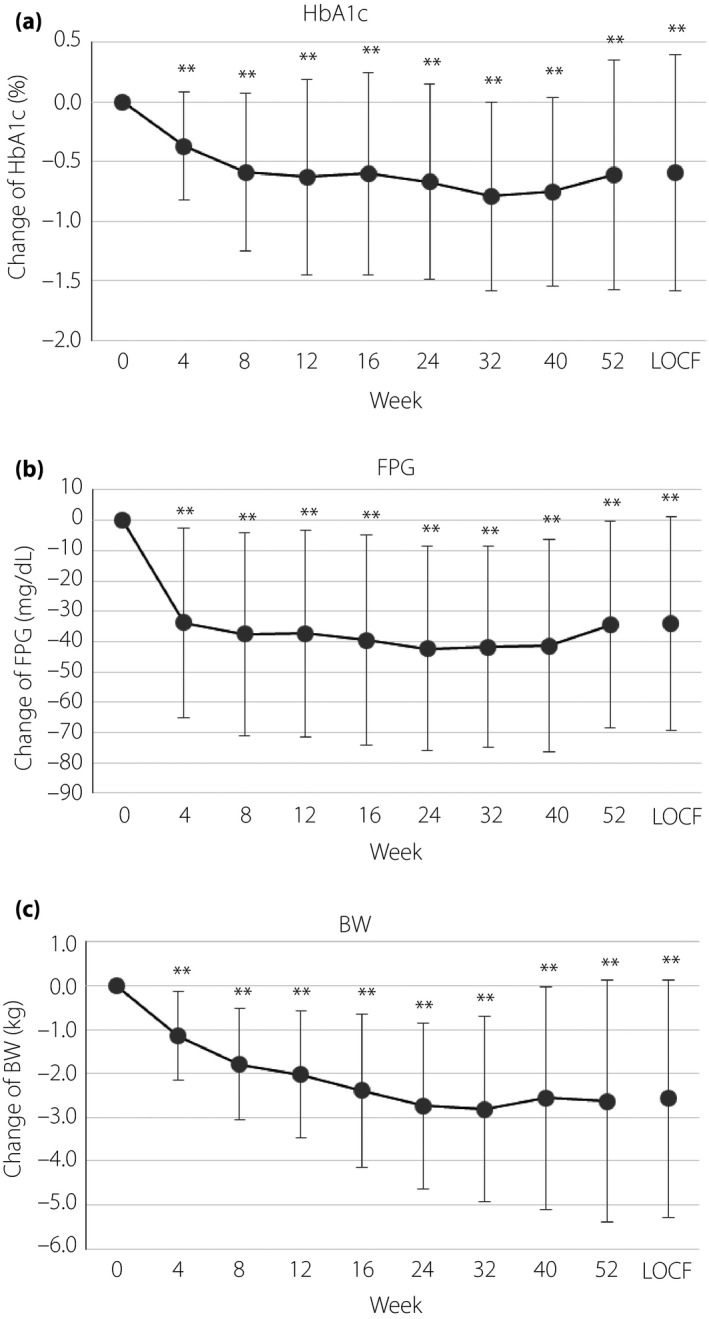
Changes from baseline to week 52 (last observation carried forward [LOCF]). (a) Glycated hemoglobin (HbA1c; %). (b) Fasting plasma glucose (FPG; mg/dL). (c) Bodyweight (BW; kg). The one sample *t*‐test, **P < 0.0001.

Regarding the primary efficacy end‐point, with the addition of tofogliflozin to GLP‐1 receptor agonists, the subsequent mean change in HbA1c from baseline to week 52 (LOCF) was −0.6% (SD 1.0%; *P *<* *0.0001; Table 3). In the secondary efficacy end‐points, the mean reduction in FPG and bodyweight from baseline to week 52 (LOCF) were −33.9 mg/dL (SD 35.2 mg/dL; *P *<* *0.0001) and −2.6 kg (SD 2.7 kg; *P *<* *0.0001), respectively (Table 3). These significant improvements, including reduction in HbA1c, were found after the first 4‐week add‐on treatment with tofogliflozin and continued through the study (Figure 1). Change in HbA1c from baseline to week 52 (LOCF) classified by patient characteristics is shown in Table S5. Bodyweight, waist circumference, SBP, DBP and TG decreased significantly, whereas significant increases in TC and HDL‐C were observed (Table S4). Glycated albumin, insulin, C‐peptide, proinsulin and homeostatic model assessment of insulin resistance were significantly reduced. There was no significant change noted in the homeostatic model assessment of β‐cell function, estimated glomerular filtration rate and urate during the observation period (Table S4).

**Table 3:** Surrogate marker of efficacy, and efficacy‐related vital signs and laboratory variables

	Baseline (week 0)	Week 52 (LOCF)		*P*‐value*
	*n* = 67	*n* = 67	Change from baseline	
HbA1c (%)	8.57 ± 1.04	7.98 ± 1.02	−0.59 ± 0.99	<0.0001
FPG (mg/dL)	190.7 ± 39.0	156.9 ± 31.3	−33.9 ± 35.2	<0.0001
Bodyweight (kg)	73.16 ± 10.33	70.59 ± 10.74	−2.57 ± 2.71	<0.0001
Systolic blood pressure (mmHg)	130.0 ± 15.4	125.9 ± 12.5	−4.1 ± 16.2	0.0422
Diastolic blood pressure (mmHg)	81.3 ± 9.8	78.4 ± 8.9	−2.9 ± 10.6	0.0290
Urate (mg/dL)	4.89 ± 1.19	4.73 ± 1.20	−0.17 ± 0.87	0.1252
Total cholesterol (mg/dL)	198.6 ± 36.9	209.8 ± 37.3	11.2 ± 27.8	0.0015
HDL cholesterol (mg/dL)	53.3 ± 14.3	58.0 ± 14.9	4.7 ± 7.1	<0.0001
LDL cholesterol (mg/dL)	122.7 ± 29.6	127.0 ± 31.4	4.3 ± 23.8	0.1410
Triglycerides (mg/dL)	166.9 ± 89.4	140.1 ± 67.1	−26.8 ± 74.1	0.0042
Free fatty acid (mEq/L)	0.646 ± 0.223	0.665 ± 0.211	0.019 ± 0.229	0.5013
Non‐HDL cholesterol (mg/dL)	145.3 ± 33.5	151.8 ± 34.0	6.5 ± 26.2	0.0462

Data presented as the mean ± standard deviation. *One sample *t*‐test of change from baseline to last observation carried forward (LOCF). FPG, fasting plasma glucose; HbA1c, glycated hemoglobin; HDL, high‐density lipoprotein; LDL, low‐density lipoprotein.

## Discussion

The present study shows that long‐term treatment with tofogliflozin for 1 year was safe and clinically useful when administered to patients with type 2 diabetes mellitus receiving GLP‐1 receptor agonist monotherapy, whose HbA1c levels had been poorly controlled. In general, many patients with type 2 diabetes mellitus often require multiple antidiabetic agents to achieve and maintain the targeted glycemic control because of disease progression due to worsening pancreatic β‐cell function and developing insulin resistance^18^. SGLT2 inhibitors have a unique mechanism of action and are independent of insulin or the degree of insulin resistance; therefore, they could be combined with any class of antidiabetic agents at any stage of disease progression in type 2 diabetes mellitus^19^, ^20^. GLP‐1 receptor agonists increase glucose‐dependent insulin secretion and suppress glucagon secretion^21^, ^22^. Both agents have been shown to improve glycemic control, and to have beneficial effects on weight loss, blood pressure and cholesterol levels with low risk of hypoglycemia^19^, ^21^. On the basis of the complementary mechanism of actions and our pervious findings, GLP‐1 receptor agonist was first selected as a concomitant antidiabetic agent among various antidiabetic agents.

The frequency of ADRs in the present study was 25.4%, which is comparable to those at approval (37.5%)^13^, ^14^ and in the post‐marketing surveillance (17.9%)^12^. Any factors including age, sex, BMI, estimated glomerular filtration rate, duration of type 2 diabetes mellitus and baseline HbA1c influencing the incidence of ADRs were not found in this study. Treatment with SGLT2 inhibitors is often associated with a risk of urinary tract and genital infections, ketoacidosis, and polyuria/pollakiuria as a class effect, but low risk of hypoglycemia^11^. ADRs of special interest observed in the present study were thirst (6.0%) and dehydration (4.5%) as a volume depletion‐related event, and pollakiuria (4.5%) as a polyuria/pollakiuria. One serious ADR, brain stem infarction, might be caused due to a volume depletion‐related event and pollakiuria. Reason was not found, but patients should be advised to take adequate hydration for preventing a volume depletion. The incidence of hypoglycemia was very limited as observed in each monotherapy.

Intensive glycemic control with tofogliflozin was shown when added to GLP‐1 receptor agonists in patients with type 2 diabetes mellitus whose HbA1c levels had been poorly controlled with GLP‐1 receptor agonist monotherapy, which is consistent with the robust efficacy reported in the previous randomized placebo‐controlled, double‐blind trials^9^, long‐term phase III clinical trial^10^ and post‐marketing surveillance in elderly patients^12^. Greater reduction in HbA1c was achieved in patients with higher baseline regardless of the combination with GLP‐1 receptor agonist, as reported^23^. Tofogliflozin also significantly reduced glycated albumin (*P *<* *0.0001), which is a marker of monitoring and screening of type 2 diabetes mellitus, as well as a predictor of long‐term outcomes of the disease^24^. Insulin, C‐peptide and proinsulin were significantly reduced throughout the present study. In contrast, there were no significant changes in glucagon and the intact proinsulin/insulin ratio, suggesting that tofogliflozin prevented the deterioration of pancreatic β‐cell function.

Tofogliflozin showed additional beneficial effects on bodyweight, blood pressure and lipid parameters. The change in bodyweight loss from baseline to week 52 (LOCF) was −2.6 kg, which is comparable to the finding in the long‐term phase III study^10^ and post‐marketing surveillance^12^. Interestingly, tofogliflozin reduced bodyweight after bodyweight loss resulting from treatment with GLP‐1 receptor agonist, which was consistently shown in 4–6‐kg bodyweight loss^25^. Furthermore, tofogliflozin significantly reduced SBP and DBP due to the osmotic diuretic effect. Mean changes from baseline in SBP (130.0 mmHg) and DBP (81.3 mmHg) over 52 weeks were −4.1 and −2.9 mmHg, respectively. The SGLT2 inhibitors were associated with a 10–15% reduction in plasma uric acid levels as a result of increased glycosuria^26^. Similarly, significant uric acids reduction was found in the previous two long‐term studies of tofogliflozin^10^, ^12^, but no significant change was observed in the present study. This difference was unclear. There were significant increases in TC and HDL‐C, and a significant decrease in TG, but no significant change in LDL‐C and FFA in the present study. The long‐term phase III study showed that 20 mg of tofogliflozin treatment caused an increase in HDL‐C and decrease in LDL‐C, but did not change TG^10^. The post‐marketing surveillance study showed that 20 mg of tofogliflozin treatment caused an increase in HDL‐C, but did not change LDL‐C and TG^12^. The effects of tofogliflozin on plasma lipid might not be straightforward as Kakuda *et al*. reported^27^, and furthermore, the addition of GLP‐1 receptor agonist would have more complex effects on the outcomes of lipids. Further study is required to evaluate the mode of action of tofogliflozin in lipid metabolism. Recent small retrospective cohort studies showed clinical benefits, including weight loss and diabetes improvement, with the combination of GLP‐1 receptor agonists and SGLT2 inhibitors^28^, ^29^. The combination of dapagliflozin with GLP‐1 receptor agonist was reported to be generally well tolerated, and significantly reduced the mean HbA1c levels and bodyweight in patients with type 2 diabetes mellitus, with a significant decrease in blood pressure, a significant increase of TC and HDL‐C, and a decrease of TG^30^ Deo *et al* 31 reported a retrospective study of combination therapy of GLP‐1 receptor agonists with SGLT2 inhibitors for the management of diabesity, resulting in significant improvements in clinical parameters, such as bodyweight loss (3.1 kg), HbA1c reduction (1.1%), lower BMI (−1.1 kg/m^2^) and insulin dose reduction (6.8 units), but the combination therapy was not associated with a reduction in blood pressure. Co‐administration of a GLP‐1 receptor agonist (exenatide) and a SGLT2 inhibitor (dapagliflozin) improved various glycemic variables and cardiovascular risk factors in patients with type 2 diabetes mellitus inadequately controlled by metformin monotherapy^32^. HbA1c reduction and weight loss in patients receiving the combination therapy, who did not respond well to metformin, was greater than those receiving either drug alone. A reduction in fasting glucose, postprandial glucose and SBP were also observed, with greater benefit than those receiving either drug alone. In contrast, our multicenter, prospective study showed that the reduction in HbA1c and weight loss in the combination of tofogliflozin and GLP‐1 receptor agonist was less than the additive effect of tofogliflozin monotherapy plus GLP‐1 receptor agonist monotherapy. However, this is the first report showing that tofogliflozin provided additional reductions in HbA1c with weight loss and reduction in blood pressure without an increase in insulin and glucagon in patients with type 2 diabetes mellitus who had been treated with GLP‐1 receptor agonist monotherapy but were inadequately controlled.

The present study had some limitations. The first was the lack of a placebo‐ and monotherapy‐controlled study. Meanwhile, the robust efficacy of tofogliflozin for the treatment of type 2 diabetes mellitus has been elucidated in a previous placebo‐controlled study^9^. Second, the proportion of elderly patients in the present study was lower than those in the real world. In a previous long‐term study of tofogliflozin in elderly patients, the concomitant use of GLP‐1 receptor agonist was a factor influencing all ADRs of special interest (odds ratio 2.90, 95% confidence interval 1.32–6.33; *P *=* *0.008) and skin complications (odds ratio 7.11, 95% confidence interval 2.28–22.15; *P *<* *0.001)^12^. However, these were not found in the present study. The number of patients in the present study was just 63, so an additional study with a high number of elderly patients is required to evaluate the safety of the combination of tofogliflozin with GLP‐1 receptor agonist. Finally, although the number of patients of 67 was decided in accordance with the Guideline for Clinical Evaluation of Oral Hypoglycemic Agents^17^, the sample size was too small to detect rare side‐effects caused by the combination of tofogliflozin with GLP‐1 receptor agonist. The safety information of tofogliflozin is limited when used in combination with other antidiabetic agents, including biguanide, sulfonylurea, dipeptidyl peptidase‐4 inhibitor, meglitinide, α‐glucosidase inhibitor, insulin sensitizer and insulin, which are commonly used as a concomitant medication in the real world. Therefore, additional post‐marketing studies are required.

In conclusion, clinically significant safety concerns have not been found in the combination of tofogliflozin with GLP‐1 receptor agonist in the present 52‐week treatment in the real world. Tofogliflozin add‐on treatment showed a long‐term reduction in HbA1c over 52 weeks, with weight loss in patients whose hyperglycemia had been inadequately controlled with a GLP‐1 receptor agonist. The combination of tofogliflozin and GLP‐1 receptor agonist is an effective treatment option with an acceptable safety profile.

## Disclosure

Yasuo Terauchi has received honoraria for speakers bureau from Astellas Pharma Inc., Astra Zeneca K.K., Bayer Yakuhin, Ltd., Daiichi Sankyo Company Limited, Dainippon Sumitomo Pharma Co., Ltd., Eli Lilly Japan K.K., Kowa Pharmaceutical Company Ltd., Merck Sharp & Dohme K.K., Mitsubishi Tanabe Pharma Corporation, Nippon Boehringer Ingelheim Co., Ltd., Novo Nordisk Pharma Ltd., Ono Pharmaceutical Co., Ltd., Sanwa Kagaku Kenkyusho Co., Ltd., Sanofi K.K., Shionogi & Co., Ltd., Taisho Toyama Pharmaceutical Co., Ltd., and Takeda Pharmaceutical Company Limited; and grants from Astellas Pharma Inc., AstraZeneca K.K., Bayer Yakuhin, Ltd., Daiichi Sankyo Company Limited, Dainippon Sumitomo Pharma Co., Ltd., Eli Lilly Japan K.K., Kowa Pharmaceutical Company Ltd., MSD K.K., Mitsubishi Tanabe Pharma Corporation, Nippon Boehringer Ingelheim Co., Ltd., Novo Nordisk Pharma Ltd., Ono Pharmaceutical Co., Ltd., Pfizer Japan Inc., Sanwa Kagaku Kenkyusho Co., Ltd., Sanofi K.K., Shionogi & Co., Ltd, Taisho Toyama Pharmaceutical Co., Ltd., and Takeda Pharmaceutical Company Limited. Ryoji Gunji, Yuji Kurihara, Hisataka Fujiwara are Hideki Suganami are employees of Kowa Company, Ltd. Masahiro Tamura and Masayuki Senda are employees of Sanofi K.K. Kohei Kaku is an advisor to, received honoraria for lectures from, and received scholarship grants from Astellas Pharma, AstraZeneca, Boehringer Ingelheim, Sumitomo Dainippon Pharma, Fujifilm Pharma, Kissei Pharmaceutical, Kowa, MSD, Novartis Pharma, Ono Pharmaceutical, Sanofi K.K., Takeda Pharmaceutical, Mitsubishi Tanabe Pharma, Taisho Toyama Pharmaceutical and Daiichi Sankyo.

## Supporting information


**Table S1** | Study sites.
**Table S2** | Adverse events of this study and at approval (safety analysis set).
**Table S3** | Adverse drug reactions in system organ class by patient characteristics.
**Table S4** | Surrogate marker of efficacy, and efficacy‐related vital signs and laboratory variables.
**Table S5** | Change in HbA1c (%) from baseline to week 52 (LOCF) classified by patient characteristics.
**Figure S1** | Patient disposition.
