# Supplementary material for: Long‐term safety and efficacy of the sodium–glucose cotransporter 2 inhibitor, tofogliflozin, added on glucagon‐like peptide‐1 receptor agonist in Japanese patients with type 2 diabetes mellitus: A 52‐week open‐label, multicenter, post‐marketing clinical study
Source: J Diabetes Investig. 2019 May 28;10(6):1518–26. doi: 10.1111/jdi.13066 (PMC6825952; doi:10.1111/jdi.13066)
Supplement: Supplementary file 1 — Table S1 | Study sites. Table S2 | Adverse events of this study and at approval (safety analysis set). Table S3 | Adverse drug reactions in system organ class by patient characteristics. Table S4 | Surrogate marker of efficacy, and efficacy‐related vital signs and laboratory variables. Table S5 | Change in HbA1c (%) from baseline to week 52 (LOCF) classified by patient characteristics. Figure S1 | Patient disposition. [file JDI-10-1518-s001.docx]

**Supplemental Tables**

**Long-term safety and efficacy of the sodium glucose cotransporter 2 inhibitor tofogliflozin added on glucagon-like peptide-1 receptor agonist in Japanese patients with type 2 diabetes mellitus (J-STEP/GLP): a 52-week open-label, multicentre, post-marketing study**

Table S1 Study sites

Table S2 Adverse events of this study and at approval (safety analysis set)

Table S3 Adverse drug reactions in system organ class by patient characteristics

Table S4 Surrogate marker of efficacy, and efficacy-related vital signs and laboratory variables

Table S5 Change in HbA1c (%) from baseline to week 52 (LOCF) classified by patient characteristics

Figure S1 Patient disposition

**Table S1 Study sites**

| Higashirinkann Kaneshiro Diabetes Clinic |
| --- |
| Tokyo-Eki Center-building Clinic |
| Fukuwa Clinic |
| Association of medical corporation Ouitsu-kai, Kanno Clinic |
| Seiwa Clinic |
| Suto Hospital |
| Wakakusa Clinic |
| Nakakinen Clinic |
| Hotaruno Central Clinic |
| Japan Organization of Occupational Health and Safety Chubu Rosai Hospital |
| Nakayama Clinic |
| Kasaoka Daiichi Hospital |

**Table S2 Adverse events of this study and at approval (safety analysis set)**

|  | This study | |  | At approval^†^ |
| --- | --- | --- | --- | --- |
| System organ class | All | ADR |  | ADR |
|  | N = 67 | |  | N = 1060 |
|  | n (%) | n (%) |  | n (%) |
| All | 38 (56.7) | 17 (25.4) |  | 397 (37.5) |
| Blood and lymphatic system disorders | 0 (0) | 0 (0) |  | 5 (0.5) |
| Cardiac disorders | 1 (1.5) | 0 (0) |  | 12 (1.1) |
| Ear and labyrinth disorders | 0 (0) | 0 (0) |  | 7 (0.7) |
| Endocrine disorders | 0 (0) | 0 (0) |  | 3 (0.3) |
| Eye disorders | 1 (1.5) | 0 (0) |  | 10 (0.9) |
| Gastrointestinal disorders | 10 (14.9) | 4 (6.0) |  | 57 (5.4) |
| General disorders and administration site conditions | 6 (9.0) | 5 (7.5) |  | 95 (9.0) |
| Hepatobiliary disorders | 1 (1.5) | 0 (0) |  | 3 (0.3) |
| Infections and infestations | 21 (31.3) | 1 (1.5) |  | 48 (4.5) |
| Injury, poisoning and procedural complications | 3 (4.5) | 0 (0) |  | 1 (0.1) |
| Investigations | 6 (9.0) | 2 (3.0) |  | 155 (14.6) |
| Metabolism and nutrition disorders | 6 (9.0) | 4 (6.0) |  | 46 (4.3) |
| Musculoskeletal and connective tissue disorders | 4 (6.0) | 0 (0) |  | 9 (0.9) |
| Neoplasms benign, malignant and unspecified (including cysts and polyps) | 0 (0) | 0 (0) |  | 3 (0.3) |
| Nervous system disorders | 4 (6.0) | 2 (3.0) |  | 23 (2.2) |
| Psychiatric disorders | 1 (1.5) | 0 (0.0) |  | 2 (0.2) |
| Renal and urinary disorders | 3 (4.5) | 3 (4.5) |  | 97 (9.2) |
| Reproductive system and breast disorders | 2 (3.0) | 2 (3.0) |  | 16 (1.5) |
| Respiratory, thoracic and mediastinal disorders | 6 (9.0) | 0 (0.0) |  | 3 (0.3) |
| Skin and subcutaneous tissue disorders | 3 (4.5) | 1 (1.5) |  | 16 (1.5) |
| Vascular disorders | 2 (3.0) | 2 (3.0) |  | 7 (0.7) |

MedDRA Version 20.0 at this study and Version 18.1 at approval

†　Utsunomiya K, Shimmoto N, Senda M et al. Safety and effectiveness of tofogliflozin in elderly Japanese patients with type 2 diabetes mellitus: A post-marketing study (J-STEP/EL Study). J Diabetes Investig. 2017. doi: 10.1111/jdi.12626.

ADR: adverse drug reaction

**Table S3 Adverse drug reactions in system organ class by patient characteristics**

|  | All | Age (years) | | Sex | | BMI (kg/m^2^) | | |
| --- | --- | --- | --- | --- | --- | --- | --- | --- |
|  |  | <65 | ≥65 | Male | Female | <25 | ≥25 to <30 | ≥30 |
| N | 67 | 58 | 9 | 45 | 22 | 19 | 36 | 12 |
| System organ class | n (%) |  |  |  |  |  |  |  |
| Gastrointestinal disorders | 4 (6.0) | 2 (3.4) | 2 (22.2) | 2 (4.4) | 2 (9.1) | 2 (10.5) | 2 (5.6) | 0 (0.0) |
| General disorders and administration site conditions | 5 (7.5) | 4 (6.9) | 1 (11.1) | 3 (6.7) | 2 (9.1) | 2 (10.5) | 2 (5.6) | 1 (8.3) |
| Infections and infestations | 1 (1.5) | 1 (1.7) | 0 (0.0) | 1 (2.2) | 0 (0.0) | 0 (0.0) | 1 (2.8) | 0 (0.0) |
| Investigations | 2 (3.0) | 2 (3.4) | 0 (0.0) | 1 (2.2) | 1 (4.5) | 0 (0.0) | 2 (5.6) | 0 (0.0) |
| Metabolism and nutrition disorders | 4 (6.0) | 4 (6.9) | 0 (0.0) | 3 (6.7) | 1 (4.5) | 1 (5.3) | 3 (8.3) | 0 (0.0) |
| Nervous system disorders | 2 (3.0) | 1 (1.7) | 1 (11.1) | 1 (2.2) | 1 (4.5) | 0 (0.0) | 1 (2.8) | 1 (8.3) |
| Renal and urinary disorders | 3 (4.5) | 2 (3.4) | 1 (11.1) | 2 (4.4) | 1 (4.5) | 0 (0.0) | 3 (8.3) | 0 (0.0) |
| Reproductive system and breast disorders | 2 (3.0) | 1 (1.7) | 1 (11.1) | 1 (2.2) | 1 (4.5) | 0 (0.0) | 1 (2.8) | 1 (8.3) |
| Skin and subcutaneous tissue disorders | 1 (1.5) | 1 (1.7) | 0 (0.0) | 1 (2.2) | 0 (0.0) | 1 (5.3) | 0 (0.0) | 0 (0.0) |
| Vascular disorders | 2 (3.0) | 1 (1.7) | 1 (11.1) | 2 (4.4) | 0 (0.0) | 1 (5.3) | 1 (2.8) | 0 (0.0) |

BMI: body mass index

MedDRA Version 20.0

**Table S3 Adverse drug reactions in system organ class by patient characteristics (Cont.)**

|  | All | eGFR (mL/min/1.73 m^2^) | | | Duration (years) | | HbA1c (%) | |
| --- | --- | --- | --- | --- | --- | --- | --- | --- |
|  |  | ≥45 to <60 | ≥60 to <90 | ≥90 | ≥1 to <10 | ≥10 | <8 | ≥8 |
| N | 67 | 6 | 26 | 35 | 38 | 29 | 22 | 45 |
| System organ class | n (%) |  |  |  |  |  |  |  |
| Gastrointestinal disorders | 4 (6.0) | 0 (0.0) | 2 (7.7) | 2 (5.7) | 3 (7.9) | 1 (3.4) | 2 (9.1) | 2 (4.4) |
| General disorders and administration site conditions | 5 (7.5) | 0 (0.0) | 3 (11.5) | 2 (5.7) | 1 (2.6) | 4 (13.8) | 3 (13.6) | 2 (4.4) |
| Infections and infestations | 1 (1.5) | 0 (0.0) | 0 (0.0) | 1 (2.9) | 1 (2.6) | 0 (0.0) | 0 (0.0) | 1 (2.2) |
| Investigations | 2 (3.0) | 0 (0.0) | 0 (0.0) | 2 (5.7) | 2 (5.3) | 0 (0.0) | 0 (0.0) | 2 (4.4) |
| Metabolism and nutrition disorders | 4 (6.0) | 0 (0.0) | 2 (7.7) | 2 (5.7) | 2 (5.3) | 2 (6.9) | 2 (9.1) | 2 (4.4) |
| Nervous system disorders | 2 (3.0) | 1 (16.7) | 1 (3.8) | 0 (0.0) | 1 (2.6) | 1 (3.4) | 1 (4.5) | 1 (2.2) |
| Renal and urinary disorders | 3 (4.5) | 0 (0.0) | 2 (7.7) | 1 (2.9) | 2 (5.3) | 1 (3.4) | 0 (0.0) | 3 (6.7) |
| Reproductive system and breast disorders | 2 (3.0) | 0 (0.0) | 0 (0.0) | 2 (5.7) | 2 (5.3) | 0 (0.0) | 1 (4.5) | 1 (2.2) |
| Skin and subcutaneous tissue disorders | 1 (1.5) | 0 (0.0) | 0 (0.0) | 1 (2.9) | 0 (0.0) | 1 (3.4) | 1 (4.5) | 0 (0.0) |
| Vascular disorders | 2 (3.0) | 0 (0.0) | 1 (3.8) | 1 (2.9) | 1 (2.6) | 1 (3.4) | 1 (4.5) | 1 (2.2) |

eGFR: estimated glomerular filtration rate, HbA1c: glycated hemoglobin

MedDRA Version 20.0

**Table S4 Surrogate marker of efficacy, and efficacy-related vital signs and laboratory variables**

|  | Week | Week 0 | Week 4 | Week 8 | Week 12 | Week 16 | Week 24 | Week 32 | Week 40 | Week 52 | LOCF | *P* value |
| --- | --- | --- | --- | --- | --- | --- | --- | --- | --- | --- | --- | --- |
|  | (n) | (67) | (66) | (66) | (66) | (64) | (63) | (63) | (63) | (63) | (67) |  |
| HbA1c (%) | |  |  |  |  |  |  |  |  |  |  |  |
|  | Mean ± SD | 8.57 ± 1.04 | 8.22 ± 0.98 | 7.99 ± 0.89 | 7.95 ± 0.91 | 8.00 ± 0.94 | 7.91 ± 0.83 | 7.78 ± 0.76 | 7.82 ± 0.81 | 7.97 ± 0.94 | 7.98 ± 1.02 |  |
|  | Change from baseline ± SD |  | -0.37 ± 0.45 | -0.59 ± 0.66 | -0.63 ± 0.82 | -0.60 ± 0.85 | -0.67 ± 0.82 | -0.79 ± 0.79 | -0.75 ± 0.79 | -0.61 ± 0.96 | -0.59 ± 0.99 | <0.0001^†^ |
| FPG (mg/dL) | |  |  |  |  |  |  |  |  |  |  |  |
|  | Mean ± SD | 190.7 ± 39.0 | 157.1 ± 33.4 | 154.2 ± 26.1 | 154.3 ± 28.3 | 152.2 ± 27.5 | 148.0 ± 25.8 | 148.5 ± 26.1 | 149.0 ± 25.3 | 155.8 ± 29.1 | 156.9 ± 31.3 |  |
|  | Change from baseline ± SD |  | -33.8 ± 31.4 | -37.5 ± 33.5 | -37.4 ± 34.2 | -39.5 ± 34.5 | -42.3 ± 33.8 | -41.8 ± 33.1 | -41.4 ± 35.0 | -34.5 ± 34.3 | -33.9 ± 35.2 | <0.0001^†^ |
| Body weight (kg) | |  |  |  |  |  |  |  |  |  |  |  |
|  | Mean ± SD | 73.16 ± 10.33 | 71.84 ± 10.19 | 71.37 ± 10.30 | 71.17 ± 10.45 | 70.96 ± 10.44 | 70.71 ± 10.67 | 70.64 ± 10.84 | 70.90 ± 10.81 | 70.82 ± 10.85 | 70.59 ± 10.74 |  |
|  | Change from baseline ± SD |  | -1.14 ± 1.02 | -1.79 ± 1.26 | -2.02 ± 1.44 | -2.39 ± 1.74 | -2.74 ± 1.89 | -2.81 ± 2.12 | -2.56 ± 2.54 | -2.63 ± 2.76 | -2.57 ± 2.71 | <0.0001^†^ |
| Waist circumference (cm) | |  |  |  |  |  |  |  |  |  |  |  |
|  | Mean ± SD | 94.26 ± 8.22 |  |  |  |  |  |  |  | 92.09 ± 7.90 | 91.98 ± 8.12 |  |
|  | Change from baseline ± SD |  |  |  |  |  |  |  |  | -2.28 ± 3.73 | -2.28 ± 3.66 | <0.0001^‡^ |
| C-peptide (μg/L) | |  |  |  |  |  |  |  |  |  |  |  |
|  | Mean ± SD | 2.00 ± 0.89 | 1.77 ± 0.76 |  | 1.78 ± 0.73 |  | 1.68 ± 0.76 | 1.69 ± 0.81 | 1.60 ± 0.64 | 1.68 ± 0.81 | 1.72 ± 0.81 |  |
|  | Change from baseline ± SD |  | -0.23 ± 0.71 |  | -0.22 ± 0.48 |  | -0.31 ± 0.49 | -0.31 ± 0.76 | -0.40 ± 0.61 | -0.31 ± 0.54 | -0.29 ± 0.54 | <0.0001^‡^ |
| Insulin (mU/L) | |  |  |  |  |  |  |  |  |  |  |  |
|  | Mean ± SD | 13.16 ± 9.80 | 9.87 ± 6.36 |  | 9.96 ± 6.04 |  | 9.65 ± 6.43 | 9.36 ± 5.67 | 8.99 ± 4.97 | 9.49 ± 6.12 | 9.87 ± 6.30 |  |
|  | Change from baseline ± SD |  | -3.20 ± 7.36 |  | -2.95 ± 6.17 |  | -3.32 ± 6.88 | -3.92 ± 7.62 | -4.18 ± 7.32 | -3.58 ± 6.72 | -3.23 ± 6.70 | <0.0001^‡^ |
| Proinsulin (pmol/L) | |  |  |  |  |  |  |  |  |  |  |  |
|  | Mean ± SD | 10.2 ± 7.3 | 8.0 ± 7.0 |  | 7.8 ± 5.8 |  | 7.0 ± 5.4 | 7.7 ± 5.6 | 7.4 ± 5.4 | 7.5 ± 5.1 | 7.7 ± 5.1 |  |
|  | Change from baseline ± SD |  | -2.2 ± 4.4 |  | -2.4 ± 3.2 |  | -3.2 ± 4.0 | -2.5 ± 4.5 | -2.8 ± 4.7 | -2.7 ± 3.9 | -2.6 ± 3.9 | <0.0001^‡^ |

**Table S4 Surrogate marker of efficacy, and efficacy-related vital signs and laboratory variables (Cont.)**

|  | Week | Week 0 | Week 4 | Week 8 | Week 12 | Week 16 | Week 24 | Week 32 | Week 40 | Week 52 | LOCF | *P* value |
| --- | --- | --- | --- | --- | --- | --- | --- | --- | --- | --- | --- | --- |
|  | (n) | (67) | (66) | (66) | (66) | (64) | (63) | (63) | (63) | (63) | (67) |  |
| Glucagon (ng/L) | |  |  |  |  |  |  |  |  |  |  |  |
|  | Mean ± SD | 96.9 ± 20.8 | 101.7 ± 26.7 |  | 100.8 ± 21.1 |  | 99.8 ± 24.5 | 96.6 ± 21.6 | 102.2 ± 22.4 | 100.8 ± 29.4 | 100.5 ± 28.7 |  |
|  | Change from baseline ± SD |  | 5.1 ± 21.7 |  | 3.6 ± 19.4 |  | 2.1 ± 22.4 | -1.0 ± 18.6 | 4.5 ± 22.3 | 3.2 ± 24.6 | 3.6 ± 24.0 | 0.5714^‡^ |
| Glycated albumin (%) | |  |  |  |  |  |  |  |  |  |  |  |
|  | Mean ± SD | 22.74 ± 4.65 | 20.27 ± 3.60 |  | 19.28 ± 3.39 |  | 19.18 ± 3.18 | 19.15 ± 2.99 | 19.45 ± 3.36 | 19.72 ± 3.52 | 19.76 ± 3.58 |  |
|  | Change from baseline ± SD |  | -2.52 ± 2.27 |  | -3.51 ± 3.17 |  | -3.46 ± 2.99 | -3.49 ± 3.05 | -3.20 ± 3.22 | -2.93 ± 3.47 | -2.98 ± 3.48 | <0.0001^‡^ |
| HOMA-β (%) | |  |  |  |  |  |  |  |  |  |  |  |
|  | Mean ± SD | 40.64 ± 33.45 | 41.30 ± 29.23 |  | 42.29 ± 29.10 |  | 42.91 ± 30.21 | 41.67 ± 27.70 | 40.18 ± 24.28 | 39.99 ± 30.64 | 41.74 ± 32.55 |  |
|  | Change from baseline ± SD |  | 1.03 ± 18.59 |  | 3.64 ± 17.92 |  | 3.36 ± 21.49 | 1.03 ± 26.95 | -0.70 ± 23.16 | -0.12 ± 22.39 | 1.53 ± 23.64 | 0.2395^‡^ |
| HOMA-R | |  |  |  |  |  |  |  |  |  |  |  |
|  | Mean ± SD | 6.19 ± 4.63 | 3.94 ± 3.07 |  | 3.81 ± 2.41 |  | 3.59 ± 2.49 | 3.50 ± 2.26 | 3.33 ± 1.96 | 3.68 ± 2.41 | 3.88 ± 2.67 |  |
|  | Change from baseline ± SD |  | -2.21 ± 4.04 |  | -2.35 ± 3.30 |  | -2.50 ± 3.40 | -2.71 ± 3.55 | -2.80 ± 3.60 | -2.42 ± 3.45 | -2.30 ± 3.40 | <0.0001^‡^ |
| Intact proinsulin/insulin ratio | |  |  |  |  |  |  |  |  |  |  |  |
|  | Mean ± SD | 0.92 ± 0.51 | 0.85 ± 0.49 |  | 0.82 ± 0.39 |  | 0.81 ± 0.49 | 0.88 ± 0.42 | 0.90 ± 0.56 | 0.89 ± 0.47 | 0.89 ± 0.47 |  |
|  | Change from baseline ± SD |  | -0.04 ± 0.34 |  | -0.11 ± 0.37 |  | -0.11 ± 0.39 | 0.00 ± 0.38 | 0.00 ± 0.51 | 0.01 ± 0.39 | -0.02 ± 0.40 | 0.7864^‡^ |
| Systolic blood pressure (mmHg) | |  |  |  |  |  |  |  |  |  |  |  |
|  | Mean ± SD | 130.0 ± 15.4 | 128.0 ± 14.6 | 125.6 ± 14.6 | 123.3 ± 14.7 | 123.5 ± 16.0 | 123.0 ± 13.3 | 122.6 ± 12.5 | 123.6 ± 12.3 | 125.7 ± 11.9 | 125.9 ± 12.5 |  |
|  | Change from baseline ± SD |  | -1.9 ± 12.0 | -4.4 ± 14.2 | -6.8 ± 13.2 | -6.9 ± 16.9 | -7.8 ± 14.0 | -8.2 ± 14.3 | -7.2 ± 13.6 | -5.0 ± 15.6 | -4.1 ± 16.2 | 0.0422^†^ |
| Diastolic blood pressure (mmHg) | |  |  |  |  |  |  |  |  |  |  |  |
|  | Mean ± SD | 81.3 ± 9.8 | 80.5 ± 10.5 | 78.9 ± 10.0 | 77.3 ± 8.7 | 78.4 ± 9.3 | 77.1 ± 9.1 | 77.2 ± 9.0 | 77.5 ± 8.5 | 78.0 ± 8.4 | 78.4 ± 8.9 |  |
|  | Change from baseline ± SD |  | -0.8 ± 8.1 | -2.5 ± 10.4 | -4.3 ± 7.5 | -3.3 ± 10.6 | -4.8 ± 7.4 | -4.8 ± 8.3 | -4.5 ± 8.6 | -3.9 ± 9.6 | -2.9 ± 10.6 | 0.0290^†^ |

**Table S4 Surrogate marker of efficacy, and efficacy-related vital signs and laboratory variables (Cont.)**

|  | Week | Week 0 | Week 4 | Week 8 | Week 12 | Week 16 | Week 24 | Week 32 | Week 40 | Week 52 | LOCF | *P* value |
| --- | --- | --- | --- | --- | --- | --- | --- | --- | --- | --- | --- | --- |
|  | (n) | (67) | (66) | (66) | (66) | (64) | (63) | (63) | (63) | (63) | (67) |  |
| Hematocrit (%) | |  |  |  |  |  |  |  |  |  |  |  |
|  | Mean ± SD | 44.40 ± 3.79 | 45.57 ± 4.03 |  | 46.38 ± 4.01 |  | 45.41 ± 4.10 | 45.81 ± 4.11 | 46.02 ± 4.07 | 45.50 ± 4.32 | 45.50 ± 4.20 |  |
|  | Change from baseline ± SD |  | 1.17 ± 1.78 |  | 1.92 ± 2.07 |  | 0.94 ± 2.17 | 1.33 ± 2.32 | 1.54 ± 2.49 | 1.02 ± 2.45 | 1.10 ± 2.40 | 0.0005^‡^ |
| Urate (mg/dL) | |  |  |  |  |  |  |  |  |  |  |  |
|  | Mean ± SD | 4.89 ± 1.19 | 4.61 ± 1.06 |  | 4.69 ± 1.05 |  | 4.80 ± 1.07 | 4.77 ± 1.22 | 4.79 ± 1.06 | 4.72 ± 1.21 | 4.73 ± 1.20 |  |
|  | Change from baseline ± SD |  | -0.28 ± 0.79 |  | -0.21 ± 0.86 |  | -0.10 ± 0.78 | -0.13 ± 0.95 | -0.11 ± 0.93 | -0.18 ± 0.85 | -0.17 ± 0.87 | 0.1252^†^ |
| eGFR (mL/min) | |  |  |  |  |  |  |  |  |  |  |  |
|  | Mean ± SD | 89.4 ± 21.9 | 87.3 ± 21.7 |  | 88.9 ± 21.8 |  | 89.3 ± 23.2 | 86.9 ± 23.1 | 87.9 ± 23.8 | 89.0 ± 23.6 | 88.9 ± 23.2 |  |
|  | Change from baseline ± SD |  | -1.8 ± 9.0 |  | -0.5 ± 8.3 |  | -0.4 ± 10.9 | -2.7 ± 9.9 | -1.7 ± 10.3 | -0.7 ± 10.2 | -0.6 ± 10.2 | 0.5185^‡^ |
| Urea Nitrogen (mg/dL) | |  |  |  |  |  |  |  |  |  |  |  |
|  | Mean ± SD | 13.1 ± 3.4 | 14.4 ± 3.3 |  | 15.2 ± 3.4 |  | 15.7 ± 4.0 | 15.5 ± 3.5 | 15.9 ± 3.2 | 15.6 ± 4.3 | 15.5 ± 4.2 |  |
|  | Change from baseline ± SD |  | 1.3 ± 3.3 |  | 2.1 ± 3.6 |  | 2.6 ± 3.7 | 2.4 ± 3.3 | 2.8 ± 3.5 | 2.5 ± 3.9 | 2.4 ± 3.9 | <0.0001^‡^ |
| Alanine Aminotransferase (U/L) | |  |  |  |  |  |  |  |  |  |  |  |
|  | Mean ± SD | 32.3 ± 18.1 | 31.0 ± 18.3 |  | 27.7 ± 15.5 |  | 27.0 ± 15.4 | 26.0 ± 12.8 | 27.9 ± 18.1 | 25.5 ± 11.4 | 25.5 ± 11.2 |  |
|  | Change from baseline ± SD |  | -1.4 ± 8.0 |  | -4.7 ± 9.7 |  | -5.6 ± 10.0 | -6.6 ± 12.3 | -4.7 ± 17.3 | -7.1 ± 13.1 | -6.8 ± 12.9 | <0.0001^‡^ |
| Aspartate Aminotransferase (U/L) | |  |  |  |  |  |  |  |  |  |  |  |
|  | Mean ± SD | 27.7 ± 13.6 | 27.1 ± 13.2 |  | 25.1 ± 10.2 |  | 25.0 ± 12.2 | 24.0 ± 9.8 | 26.3 ± 15.1 | 24.3 ± 9.4 | 24.2 ± 9.2 |  |
|  | Change from baseline ± SD |  | -0.6 ± 7.2 |  | -2.6 ± 7.7 |  | -3.0 ± 8.0 | -4.0 ± 8.8 | -1.7 ± 12.1 | -3.7 ± 9.0 | -3.5 ± 8.8 | 0.0042^‡^ |
| Gamma Glutamyl Transferase (U/L) | |  |  |  |  |  |  |  |  |  |  |  |
|  | Mean ± SD | 50.0 ± 39.3 | 42.7 ± 35.7 |  | 39.8 ± 30.7 |  | 41.4 ± 32.6 | 39.3 ± 29.5 | 41.5 ± 36.3 | 40.3 ± 33.8 | 39.3 ± 33.0 |  |
|  | Change from baseline ± SD |  | -7.7 ± 13.3 |  | -10.3 ± 18.9 |  | -9.7 ± 20.1 | -11.8 ± 22.0 | -9.6 ± 21.9 | -10.9 ± 15.1 | -10.7 ± 14.8 | <0.0001^‡^ |

**Table S4 Surrogate marker of efficacy, and efficacy-related vital signs and laboratory variables (Cont.)**

|  | Week | Week 0 | Week 4 | Week 8 | Week 12 | Week 16 | Week 24 | Week 32 | Week 40 | Week 52 | LOCF | *P* value |
| --- | --- | --- | --- | --- | --- | --- | --- | --- | --- | --- | --- | --- |
|  | (n) | (67) | (66) | (66) | (66) | (64) | (63) | (63) | (63) | (63) | (67) |  |
| Total cholesterol (mg/dL) | |  |  |  |  |  |  |  |  |  |  |  |
|  | Mean ± SD | 198.6 ± 36.9 | 200.9 ± 34.2 |  | 204.0 ± 34.6 |  | 205.5 ± 37.5 | 203.7 ± 34.7 | 207.1 ± 36.8 | 210.7 ± 38.1 | 209.8 ± 37.3 |  |
|  | Change from baseline ± SD |  | 1.6 ± 22.6 |  | 5.0 ± 26.1 |  | 6.1 ± 25.1 | 4.4 ± 25.3 | 7.7 ± 26.6 | 11.3 ± 28.6 | 11.2 ± 27.8 | 0.0015^†^ |
| HDL cholesterol (mg/dL) | |  |  |  |  |  |  |  |  |  |  |  |
|  | Mean ± SD | 53.3 ± 14.3 | 54.5 ± 13.4 |  | 55.6 ± 13.3 |  | 56.4 ± 14.5 | 56.8 ± 15.3 | 58.3 ± 15.0 | 58.2 ± 15.2 | 58.0 ± 14.9 |  |
|  | Change from baseline ± SD |  | 1.0 ± 5.3 |  | 2.3 ± 6.5 |  | 2.9 ± 7.4 | 3.3 ± 8.5 | 4.8 ± 8.4 | 4.7 ± 7.1 | 4.7 ± 7.1 | <0.0001^†^ |
| LDL cholesterol (mg/dL) | |  |  |  |  |  |  |  |  |  |  |  |
|  | Mean ± SD | 122.7 ± 29.6 | 125.5 ± 29.4 |  | 126.2 ± 30.9 |  | 125.3 ± 31.4 | 122.4 ± 29.5 | 125.8 ± 31.6 | 127.1 ± 32.0 | 127.0 ± 31.4 |  |
|  | Change from baseline ± SD |  | 2.3 ± 18.6 |  | 3.1 ± 21.8 |  | 2.7 ± 21.3 | -0.2 ± 23.3 | 3.2 ± 23.7 | 4.5 ± 24.5 | 4.3 ± 23.8 | 0.1410^†^ |
| Triglycerides (mg/dL) | |  |  |  |  |  |  |  |  |  |  |  |
|  | Mean ± SD | 166.9 ± 89.4 | 145.1 ± 64.7 |  | 153.2 ± 75.2 |  | 165.9 ± 152.3 | 151.2 ± 116.0 | 145.7 ± 86.9 | 140.3 ± 68.2 | 140.1 ± 67.1 |  |
|  | Change from baseline ± SD |  | -21.8 ± 68.4 |  | -14.0 ± 74.8 |  | -3.8 ± 106.2 | -18.5 ± 123.5 | -24.0 ± 82.9 | -29.4 ± 75.1 | -26.8 ± 74.1 | 0.0042^†^ |
| Free fatty acid (mEq/L) | |  |  |  |  |  |  |  |  |  |  |  |
|  | Mean ± SD | 0.646 ± 0.223 | 0.720 ± 0.257 |  | 0.677 ± 0.196 |  | 0.719 ± 0.225 | 0.658 ± 0.228 | 0.710 ± 0.232 | 0.664 ± 0.191 | 0.665 ± 0.211 |  |
|  | Change from baseline ± SD |  | 0.073 ± 0.292 |  | 0.033 ± 0.240 |  | 0.067 ± 0.273 | 0.007 ± 0.281 | 0.058 ± 0.250 | 0.013 ± 0.227 | 0.019 ± 0.229 | 0.5013^†^ |
| Non HDL cholesterol (mg/dL) | |  |  |  |  |  |  |  |  |  |  |  |
|  | Mean ± SD | 145.3 ± 33.5 | 146.4 ± 31.8 |  | 148.5 ± 33.0 |  | 149.0 ± 37.5 | 146.9 ± 33.1 | 148.8 ± 35.0 | 152.5 ± 34.8 | 151.8 ± 34.0 |  |
|  | Change from baseline ± SD |  | 0.6 ± 20.7 |  | 2.7 ± 23.3 |  | 3.2 ± 23.6 | 1.1 ± 23.6 | 3.0 ± 25.3 | 6.7 ± 27.0 | 6.5 ± 26.2 | 0.0462^†^ |
| Adiponectin (mg/L) | |  |  |  |  |  |  |  |  |  |  |  |
|  | Mean ± SD | 7.51 ± 4.04 | 7.55 ± 4.10 |  | 7.93 ± 4.36 |  | 8.12 ± 4.16 | 7.87 ± 3.68 | 8.11 ± 4.16 | 8.37 ± 4.50 | 8.23 ± 4.41 |  |
|  | Change from baseline ± SD |  | 0.01 ± 1.21 |  | 0.40 ± 1.39 |  | 0.48 ± 1.28 | 0.23 ± 1.42 | 0.47 ± 1.39 | 0.72 ± 1.64 | 0.73 ± 1.61 | <0.0001^‡^ |

†.One sample t-test of change from baseline to LOCF, ‡.the Wilcoxon signed-rank test of change from baseline to LOCF.

LOCF: last observation carried forward , SD: standard deviation, HbA1c: glycated hemoglobin, FPG: fasting plasma glucose, HOMA-β: homeostatic model assessment of beta cell function, HOMA-R: homeostasis model assessment of insulin resistance ratio, eGFR: estimated glomerular filtration rate, HDL: high-density lipoprotein, LDL: low-density lipoprotein

**Table S5 Change in HbA1c (%) from baseline to week 52 (LOCF) classified by patient characteristics**


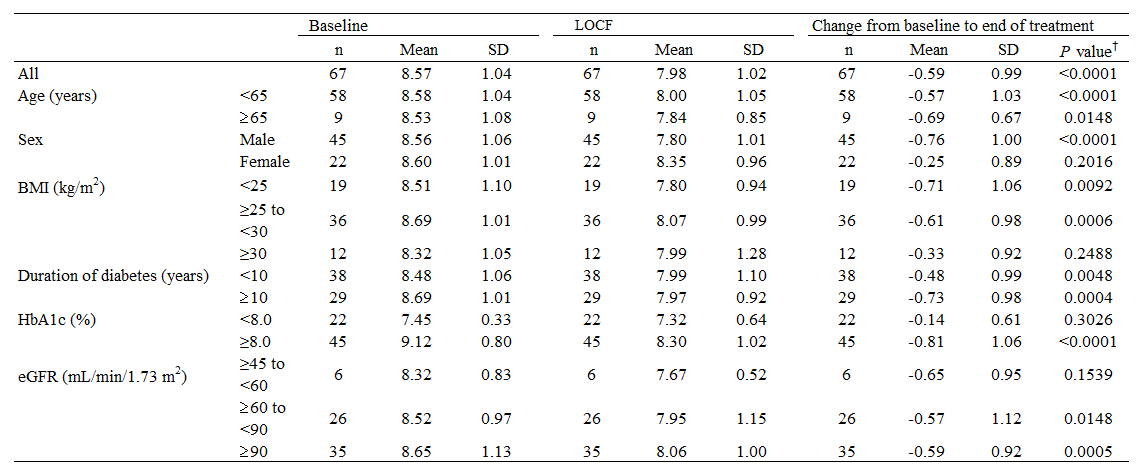


†. One sample t-test of change from baseline to end of treatment

BMI: body mass index, eGFR: estimated glomerular filtration rate, HbA1c: glycated hemoglobin, LOCF: last observation carried forward, SD: standard deviation

| Informed consent n = 73 | |  |  |  |
| --- | --- | --- | --- | --- |
|  |  |  | Withdrawal during observation period: n = 6 | |
|  |  |  |  |  |
| Patient registered n = 67 | |  |  |  |
|  |  |  | Discontinued: n = 4  Consent withdrawn (3)  Investigator's decesion (1) | |
|  |  |  |  |  |
| Completed n = 63 | |  |  |  |

**Figure S1 Patient disposition**
